# Association between childhood trauma and medication adherence among patients with major depressive disorder: the moderating role of resilience

**DOI:** 10.1186/s12888-022-04297-0

**Published:** 2022-10-14

**Authors:** Hongqiong Wang, Yuhua Liao, Lan Guo, Huimin Zhang, Yingli Zhang, Wenjian Lai, Kayla M. Teopiz, Weidong Song, Dongjian Zhu, Lingjiang Li, Ciyong Lu, Beifang Fan, Roger S. McIntyre

**Affiliations:** 1 Department of Medical Statistics and Epidemiology, School of Public Health, Sun Yat-Sen University, 74 Zhongshan Rd 2, Guangzhou, 510080 People’s Republic of China; 2 Guangdong Provincial Key Laboratory of Food, Nutrition and Health, Sun Yat-Sen University, Guangzhou, People’s Republic of China; 3 Guangdong Engineering Technology Research Center of Nutrition Translation, Guangzhou, People’s Republic of China; 4 Department of Psychiatry, Shenzhen Nanshan Center for Chronic Disease Control, 7Huaming road, Shenzhen, 518000 People’s Republic of China; 5 Department of Depressive Disorder, Shenzhen Kangning Hospital, Shenzhen Mental Health Center, Shenzhen, People’s Republic of China; 6 Institute of Medical Science, University of Toronto, Toronto, ON M5S 1A8 Canada; 7 Mental Health Institute of the Second Xiangya Hospital, Central South University, Changsha, People’s Republic of China; 8 Mood Disorders Psychopharmacology Unit, University Health Network, Toronto, ON M5T 2S8 Canada

**Keywords:** Childhood trauma, Medication adherence, Resilience, Major depressive disorder, Physical neglect, Sexual abuse

## Abstract

**Background:**

Suboptimal medication adherence is a major reason for failure in the management of major depressive disorder (MDD), childhood trauma might be an essential risk factor of suboptimal medication adherence. This study aimed to comprehensively explore the associations between different types of childhood trauma and medication adherence among patients with MDD, and to test whether resilience has moderating effects on the foregoing associations.

**Methods:**

Participants were from the Depression Cohort in China (ChiCTR registry number 1900022145), 282 MDD patients with completed both baseline and 12-weeks follow-up investigations were included in this study. The diagnosis of MDD was assessed by trained psychiatrists using the Mini-International Neuropsychiatric Interview (M.I.N.I.). Childhood trauma was evaluated using the Childhood Trauma Questionnaire-28 item Short Form (CTQ-SF), and resilience was evaluated using the Connor-Davidson Resilience Scale (CD-RISC). Demographic characteristics, depression symptoms, anxiety symptoms, suicidal ideation, suicidal attempt, insomnia symptoms, and painful somatic symptoms were also investigated. Participants were divided into groups of optimal and suboptimal adherence based on their Medication Adherence Rating Scale scores. Logistic regression and stratified analyses were performed.

**Results:**

A total of 234 participants (83%) reported suboptimal medication adherence. After adjusting for covariates, CTQ total scores (*AOR* = 1.03, 95%*CI* = 1.01–1.06), CTQ measures of sexual abuse (*AOR* = 1.17, 95%*CI* = 1.01–1.37), and CTQ measures of physical neglect (*AOR* = 1.12, 95%*CI* = 1.02–1.23) were all associated with an increased likelihood of suboptimal adherence. There were significant moderating effects of resilience on the associations of childhood trauma (*P* = 0.039) and physical neglect (*P* = 0.034) with medication adherence. The stratification analyses showed that CTQ total scores and CTQ measures of physical neglect were independently associated with an increased risk of suboptimal adherence among patients with MDD with low-resilience or moderate-resilience, while not significantly associated with suboptimal adherence in those with high-resilience.

**Conclusion:**

Childhood trauma was a significant risk factor of suboptimal adherence among patients with MDD, and resilience moderated the foregoing association. Obtaining a history of childhood trauma and assessing resilience may help identify patients with suboptimal adherence when providing MDD pharmacotherapy. Psychiatrists may consider enhancing resilience to cope with the adverse effects of childhood trauma on medication adherence.

**Supplementary Information:**

The online version contains supplementary material available at 10.1186/s12888-022-04297-0.

## Introduction

Major depressive disorder (MDD) is the most prevalent and disabling mental disorder, the global prevalence of MDD in 2019 is 2.49% [1]. Antidepressants is the first-line treatment for depression in primary care settings, as recommended by the American Psychiatric Association [2]. However, medication adherence in patients with MDD is usually very poor, with rates of suboptimal adherence ranging from 50.7% to 85.4% [3–5].

Medication adherence has been defined as “the extent to which patients take medications as prescribed by their health care providers” [6]. The most common reasons for poor medication adherence included worrying about the side effects of long‐term medication, questioning the need for long‐term medication, and assuming that their symptoms are cured [7]. Suboptimal adherence to antidepressants is widely recognized as one of the major reasons for failure in the management of MDD [8], contributing to severe symptoms, treatment-resistance, increased risk of relapse, higher disability and suicide rates, and economic burden [9–11]. Thus, improvement of medication adherence is an essential issue for the management of MDD.

Significant evidence suggests that childhood trauma increase the risk of MDD and the frequency of comorbidities [12–14]. Among the MDD population diagnosed by Structured Clinical Interviews for DSM-IV (SCID-I), 57.1% of patients had suffered from childhood trauma [15], and the population attributable risk (PAR) of MDD from childhood trauma was 54% [16]. Moreover, it has been reported that childhood trauma predicts an unfavorable course of illness and treatment outcome in people with MDD, with it being most strongly associated with an elevated risk of developing recurrent and persistent depressive episodes, and lack of response or remission during treatment [17]. Five types of childhood trauma have been distinguished: physical abuse, emotional abuse, sexual abuse, and physical as well as emotional neglect [15, 18]. In addition, early childhood trauma has been reported to contribute to either self-regulatory disturbances or a self-defeating pattern in some affected individuals, with subsequent impact to their health care adherence [19]. The association between childhood trauma and medication adherence has been explored in clinical samples such as individuals with HIV, children who had a transplant, and women treated with methadone among others [20–22]. However, few studies have explored the association between different types of childhood trauma and medication adherence among patients with MDD, this merits more research attention.

Resilience is defined as the ability to adapt to difficult situations and provide flexible responses to daily stress [23], and it is considered as an important process in the experience and management of MDD. Patients with depressive disorders exhibit lower levels of resilience, compared to healthy population or other select mental disorders [24]. For patients with MDD who have higher level of resilience, it has been observed that positive attitudes toward antidepressants had facilitated their medication adherence, whereas lower levels of resilience are associated with suboptimal adherence [25, 26].

Exposure to childhood trauma may negatively interact with pharmacotherapy in patients with MDD [27], as well as increase the risk of suboptimal adherence. However, it has been reported that some patient with history of childhood trauma and satisfactory resilience can still achieve positive treatment outcomes [28]. Thus, we hypothesize that resilience might be a significant factor for protecting against reduced medication adherence in individuals with MDD who have experienced significant childhood trauma. The study herein aimed to comprehensively explore the associations between different types of childhood trauma and medication adherence among patients with MDD, and to test whether resilience has moderating effects on the foregoing associations. Our results may inform early detection and timely intervention for patients with suboptimal medication adherence when providing MDD pharmacotherapy.

## Methods

### Study design and participants

Data were derived from the Depression Cohort in China (DCC) study (ChiCTR registry number: 1900022145) between January 2020 and September 2021, a detailed description of the DCC study design has been described elsewhere [29–31].

**Fig. 1:**
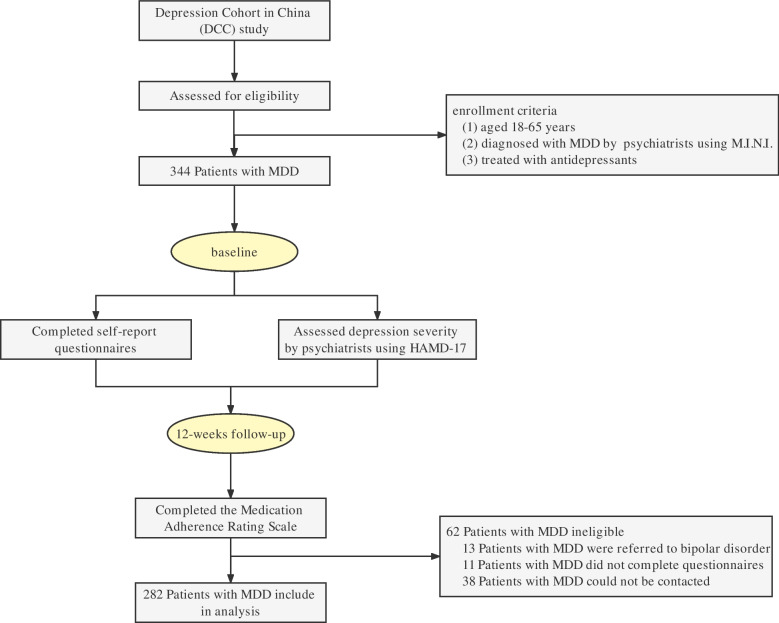
Study flow chart

Study flow chart is shown in Fig. 1. Participants were individuals aged 18–65 years, who were diagnosed with MDD by trained psychiatrists using the Mini-International Neuropsychiatric Interview (M.I.N.I.), and prescribed with antidepressant as treatment strategy by psychiatrists. At baseline, participants completed self-report questionnaires that collect information on demographic characteristics, health status and behaviors, childhood trauma, and resilience. The follow-up period was 12 weeks. At 12-week follow-up, information about medication adherence was collected. This study received ethical approval from the institutional review board of Sun Yat-sen University School of Public Health (Ethical code: L2017044), and the study protocol was approved by the Ethical Review Boards of all the participating centers. All participants signed informed consent before entering the study.

A total of 344 patients with MDD were enrolled in the study, among which 282 (81.98%) were eligible and completed the 12-weeks follow up (62 participants were excluded due to changing the diagnosis of MDD, *n* = 13; lost during follow-up, including did not complete the follow-up questionnaire and unable to contact, *n* = 49).

### Measures

#### Ascertainment of MDD and depression severity

The M.I.N.I. was used by psychiatrists to diagnose a current MDD and exclude other diagnoses according to the DSM-IV [32]. The M.I.N.I. is a validated structured diagnostic psychiatric interview by clinicians, and this instrument has shown good psychometric characteristics [33]. Depression severity was measured using the 17-item Hamilton Rating Scale for Depression (HAMD-17), which is a clinician-rated instrument developed to quantify the severity of depression in subjects already diagnosed with this disorder (Cronbach’s alpha = 0.70 in this study) [34]. The seventeen items’ total score ranges from 0 to 53, with the higher scores indicating more severe depression symptomatology [35].

#### Medication adherence

Medication adherence was assessed with the Chinese version of the Medication Adherence Rating Scale (MARS) [36]. The MARS is a self-questionnaire including 10 items yes/no combined with the Drug Attitude Inventory (DAI) [37] and Medication Adherence Questionnaire (MAQ) [38].

The MARS has good reliability and validity among the psychiatric patients [39] and showed high internal consistency (Cronbach’s alpha = 0.71 in this study) [40]. Three factors can be distinguished from MARS, factor 1 explores medication adherence behavior (items 1–4; i.e., “I sometimes forget to take my medication”; “I'm careless at times about taking my medication”; “If I feel better, sometimes I stop the treatment”; “Sometimes if I feel worse when I take my medicine, I stop taking it”), factor 2 explores beliefs to taking medication (items 5–8; i.e., “I take my medication only when I am sick”; “It's not natural for my body and mind to be balanced by drugs”; “My ideas are clearer with medication”; “By staying on medication, I can prevent getting sick”), factor 3 explores the negative side effects of psychotropics (items 9: “With the drugs I feel weird, like a zombie” and 10: “The drugs make me heavy and tired”) [41]. The MARS is scored by attributing one point to the adherent responses which are “No” for items 1–6 and 9–10, and “Yes” for items 7–8, a cutoff value of 6 was used to divide participants with MDD into optimal and suboptimal adherence groups in this study [42].

#### Childhood trauma

Childhood trauma was evaluated using the Chinese version of the Childhood Trauma Questionnaire-28 item Short Form (CTQ-SF). The CTQ-SF is a frequently used tool for assessing early traumatic experiences [43], with good reliability and high internal consistency (Cronbach’s alpha = 0.84 in this study) [44]. The scale contains five subscales to assess five types of childhood trauma: physical abuse, emotional abuse, sexual abuse, and physical as well as emotional neglect. Each subscale contains five questions, and participants were asked to rate the frequency of childhood trauma on a 5-point scale (from 1 = “never” to 5 = “always”). The overall scale had scores ranging from 25 to 125. The higher the scores, the more severe the childhood trauma.

#### Resilience

Resilience was measured using the Connor-Davidson Resilience Scale (CD-RISC, Cronbach’s alpha = 0.91 in this study), comprising 25 items with each rated on a 5-point scale ranging (from 0 = “not at all true” to 4 = “true nearly all of the time”) [45]. The CD-RISC yields a total resilience score ranging from 0 to 100, with higher scores indicating greater resilience [46].

#### Covariates

Factors associated with medication adherence or childhood trauma were taken into consideration among patients with MDD. The covariates included demographic characteristics, health status, and behaviors.

Demographic characteristics included age, sex, marital status, education level, living arrangement, and household socioeconomic status (HSS). Education level was defined as the highest degree a participant had received at the time of diagnosis of MDD, living arrangement was assessed according to the answer about whom they lived with, HSS was measured by their per capita monthly household income.

Health status and behaviors included current cigarette smoking behavior, current alcohol drinking behavior, exercise habit per week, depression severity, anxiety severity, suicidal ideation, suicidal attempt, insomnia symptoms, and painful somatic symptoms. Except for depression severity, other covariates were measured using self-report questionnaires. Current cigarette smoking behavior, current alcohol drinking behavior and exercise habit per week was assessed by inquiring the following question: “In the past 30 days, how many days have you smoked a cigarette? how many days have you had at least one alcoholic drink? have you ever exercised at least once a week for more than 30 min?”. Anxiety severity was measured using the Generalized Anxiety Disorder Scale-7 (GAD-7, Cronbach’s alpha = 0.90 in this study), which has been validated and extensively utilized in Chinese studies with satisfactory psychometric properties [47]. The total score ranges from 0 to 21, with higher scores indicating more severe anxious symptomatology. The assessment of suicidal ideation and suicide attempts use the following questions: “During the past year, how many times did you seriously consider attempting suicide?” for suicidal ideation and “During the past year, how many times did you actually attempt suicide?” for suicide attempts. Insomnia symptoms were assessed using the Insomnia Severity Index (ISI, Cronbach’s α = 0.90 in this study), which consists of 7 items, with each item scored from 0 to 4, for a maximum of 28 points [48]. Higher scores represent greater insomnia levels where participants were classified based on their score (i.e., group of none, subthreshold, moderate and severe of score 0–7, 8–14, 15–21, and 22–28, respectively) [49]. The mean score for the seven pain-related items (items 2, 3, 9, 14, 19, 27, and 28) in the 28-item Somatic Symptoms Inventory (SSI, Cronbach’s α = 0.90 in this study) was used to assess painful somatic symptoms [50]. Participants with mean scores for each item < 2.2 in SSI were considered to be without pain [51].

### Statistical analysis

First, data were described as the number [percentage (%)] for categorical variables, and as the mean [standard deviation (*SD*)] or median [interquartile range (*IQR*)] for continuous variables. Chi-square tests for categorical variables and the *t*-test and Wilcoxon’s rank-sum tests for continuous variables were used to compare optimal and suboptimal adherence groups.

Second, univariate logistic regression models were used to assess the independent association of different types of childhood trauma and resilience with medication adherence. Covariates which were significant in univariate analysis or extensively reported in the literature were selected as potential confounders. Then multivariable logistic regression models that adjusted for potential confounders were conducted to examine the independent association of childhood trauma and resilience with medication adherence, and the adjusted odds ratios (*AOR*s) and 95% confidence intervals (*CI*s) were acquired.

Third, to investigate whether the association between childhood trauma and medication adherence varied by the MDD patients’ resilience, multiplicative interaction items were tested first by entering a cross-product term for childhood trauma and resilience variable along with the main effect terms for each to the multivariable multinomial logistic regression models, and *P* values for the interaction items were calculated. If the association between the interaction item and medication adherence was statistically significant, the stratification analyses would be conducted to assess the association between childhood trauma and medication adherence by resilience among patients with MDD.

All statistical analyses were conducted using IBM SPSS Statistics 26.0 (IBM, Armonk, NY, USA), and all statistical tests were two-sided, with a *P* value less than 0.05 considered statistically significant.

## Results

### Sample characteristics of 282 patients with MDD

The distribution of baseline characteristics was balanced between eligible participants and excluded participants (eTable 1 in the supplement).

Sample characteristics are presented in Table 1. The median (IQR) age of the patients was 25.0 (9.0) years. Of the total baseline sample, 66.3% were female, 73.2% were unmarried, 55.1% reported their education level as college or above, 60.9% living with families, and 41.6% reported their HSS as excellent or very good.

**Table 1:** Sample characteristics of 282 patients with MDD

Variables	Total, *n* (%)	Optimal adherence	Suboptimal adherence	*P* Value^#^
Total	282 (100)	48 (17.0)	234 (83.0)	
Age (years), median (*IQR*)	25.0 (9.0)	26.0 (9.0)	24.0 (10.0)	0.288
Sex				
Male	95 (33.7)	13 (27.1)	82 (35.0)	0.288
Female	187 (66.3)	35 (72.9)	152 (65.0)	
Marital status				
Unmarried	199 (73.2)	35 (74.5)	164 (72.9)	0.233
Married	61 (22.4)	8 (17.0)	53 (23.6)	
Divorced/Widowed	12 (4.4)	4 (8.5)	8 (3.6)	
Missing data	10			
Education level				
Junior high school or below	50 (18.1)	16 (34.0)	34 (14.8)	** < 0.001**
Senior high school	74 (26.8)	16 (34.0)	58 (25.3)	
College or above	152 (55.1)	15 (31.9)	137 (59.8)	
Missing data	6			
Living arrangement				
Living alone	61 (22.3)	10 (21.3)	51 (22.5)	0.664
Living with families	167 (60.9)	27 (57.4)	140 (61.7)	
Living with others	46 (16.8)	10 (21.3)	36 (15.9)	
Missing data	8			
HSS				
Excellent or very good	106 (41.6)	18 (43.9)	88 (41.1)	0.870
Good	73 (28.6)	10 (24.4)	63 (29.4)	
Fair	76 (29.8)	13 (31.7)	63 (29.4)	
Missing data	27			
Current cigarette smoking				
No	130 (47.3)	26 (56.5)	104 (45.4)	0.169
Yes	145 (52.7)	20 (43.5)	125 (54.6)	
Missing data	7			
Current alcohol drinking				
No	237 (84.9)	41 (89.1)	196 (84.1)	0.385
Yes	42 (15.1)	5 (10.9)	37 (15.9)	
Missing data	3			
Exercise habit per week				
No	171 (63.1)	29 (64.4)	142 (62.8)	0.838
Yes	100 (36.9)	16 (35.6)	84 (37.2)	
Missing data	11			
HAMD-17 scores, mean (*SD*)	17.84 (5.02)	17.34 (5.03)	20.19 (4.28)	** < 0.001**
GAD-7 scores, median (*IQR*)	14.0 (8.0)	13.0 (9.0)	17.0 (5.0)	**0.005**
Suicidal ideation				
No	192 (68.8)	24 (51.1)	168 (72.4)	**0.004**
Yes	87 (31.2)	23 (48.9)	64 (27.6)	
Missing data	3			
Suicidal attempt				
No	249 (88.9)	35 (72.9)	214 (92.2)	** < 0.001**
Yes	31 (11.1)	13 (27.1)	18 (7.8)	
Missing data	2			
Insomnia				
None	40 (14.3)	3 (6.3)	37 (16.0)	**0.020**
Subthreshold	85 (30.5)	9 (18.8)	76 (32.9)	
Moderate	109 (39.1)	24 (50.0)	85 (36.8)	
Severe	45 (16.1)	12 (25.0)	33 (14.3)	
Missing data	3			
SSI pain-related items				
Without pain	90 (32.0)	25 (52.1)	65 (27.9)	** < 0.001**
With pain	192 (68.0)	23 (47.9)	168 (72.1)	
CD-RISC scores, median (*IQR*)	37.0 (18.0)	38.0 (18.0)	31.0 (14.0)	**0.001**
CTQ total scores, median (*IQR*)	46.0 (16.0)	45.0 (17.0)	49.0 (15.0)	**0.012**
CTQ scores of physical abuse, median (*IQR*)	6.0 (3.0)	5.0 (2.0)	7.0 (3.0)	**0.031**
CTQ scores of emotional abuse, median (*IQR*)	9.0 (5.0)	9.0 (6.0)	10.0 (3.0)	0.082
CTQ scores of sexual abuse, median (*IQR*)	5.0 (0.0)	5.0 (0.0)	5.0 (1.0)	**0.015**
CTQ scores of physical neglect, median (*IQR*)	8.0 (5.0)	8.0 (5.0)	10.0 (7.0)	**0.006**
CTQ scores of emotional neglect, median (*IQR*)	15.0 (8.0)	15.0 (8.0)	17.0 (8.0)	0.088

Abbreviations: *SD* Standard, *IQR* interquartile range, *HSS* Household socioeconomic status, *HAMD-17* 17-item hamilton rating scale for depression, *GAD-7* the generalized anxiety disorder scale-7, *SSI* Somatic symptoms inventory, *CD-RISC* Connor-davidson resilience scale, *CTQ* Childhood trauma questionnaire-28 item short form

^#^Chi-squared tests were used for categorical variables, *t* test was used for HAMD-17 scores data, and Wilcoxon’s rank-sum tests were used for other continuous data

Forty-eight patients (17.0%) who scored 6 or above on the MARS questionnaire at 12-weeks follow-up were categorized as the “optimal adherence group”, and the remaining participants were “suboptimal adherence group”. There were statistically significant differences between patients with suboptimal and optimal adherence in the distribution of education level (*P* < 0.01), depression severity (*P* < 0.001), anxiety severity (*P* < 0.05), suicidal ideation (*P* < 0.05), suicidal attempt (*P* < 0.001), insomnia symptoms(*P* < 0.05), and painful somatic symptoms (*P* < 0.001). In addition, compared with optimal adherence group, the total scores for CTQ (*P* < 0.05), CTQ scores of physical abuse (*P* < 0.05), CTQ scores of sexual abuse (*P* < 0.05), CTQ scores of physical neglect (*P* < 0.05) were higher, and CD-RISC scores were lower (*P* < 0.05) among suboptimal adherence group.

The most common reasons for suboptimal medication adherence are presented eTable 2 in the supplement. Based on their response on the MARS, of the suboptimal adherence group, 88.5% response “Yes” for items 4: sometimes if I feel worse when I take my medication, I stop taking it; 82.1% response “Yes” for items 9: with the drugs I feel weird, like a zombie.

### Association of childhood trauma and resilience with medication adherence

As shown in Table 2, in the univariate unadjusted models, childhood trauma, including physical abuse, sexual abuse, physical neglect, and resilience were all associated with an increased risk of suboptimal adherence (all *P* < 0.05). After adjusting for selected covariates (including age, sex, marital status, education level, HAMD-17 scores, GAD-7 scores, suicidal ideation, suicidal attempt, insomnia, SSI pain-related items), the final multivariable logistic regression analyses demonstrated that CTQ total scores (*AOR* = 1.03, 95%*CI* = 1.01–1.06), CTQ scores of sexual abuse (*AOR* = 1.17, 95%*CI* = 1.01–1.37), and CTQ scores of physical neglect (*AOR* = 1.12, 95%*CI* = 1.02–1.23) were all associated with an increased risk of suboptimal adherence. Moreover, CD-RISC scores were associated with a decreased risk of suboptimal adherence.

**Table 2:** Association between Childhood Trauma and Resilience with Medication Adherence among MDD (*N* = 282)

Variables	Univariate models		Multivariable models			
	Suboptimal adherence		Suboptimal adherence ^a^		Suboptimal adherence ^b^	
	*OR* (95%*CI*)	*P* Value	*AOR* (95%*CI*)	*P* Value	*AOR* (95%*CI*)	*P* Value
CTQ total scores^#^	1.03 (1.01–1.06)	**0.007**	1.03 (1.01–1.06)	**0.019**	NA	NA
CTQ scores of physical abuse^#^	1.09 (1.01–1.18)	**0.030**	NA	NA	1.08 (0.95–1.22)	0.241
CTQ scores of emotional abuse^#^	1.03 (0.97–1.10)	0.327	NA	NA	0.93 (0.83–1.04)	0.220
CTQ scores of sexual abuse^#^	1.17 (1.03–1.17)	**0.016**	NA	NA	1.17 (1.01–1.37)	**0.042**
CTQ scores of physical neglect^#^	1.13 (1.04–1.22)	**0.005**	NA	NA	1.12 (1.02–1.23)	**0.016**
CTQ scores of emotional neglect^#^	1.06 (0.99–1.13)	0.074	NA	NA	1.03 (0.94–1.14)	0.548
CD-RISC scores^#^	0.96 (0.94–0.98)	**0.002**	0.97 (0.95–0.99)	**0.039**	0.97 (0.94–0.99)	**0.038**

Abbreviations: *OR* Odds ratio, *AOR* Adjusted odds ratio, *CI* Confidence interval, *NA* Not applicable or not available, *CTQ* Childhood trauma questionnaire-28 item short form, *CD-RISC* Connor-davidson resilience scale

^#^For every 1-score increase in total CTQ scores, CTQ scores of physical abuse, CTQ scores of emotional abuse, CTQ scores of sexual abuse, CTQ scores of physical neglect, CTQ scores of emotional neglect, and CD-RISC scores

Univariate models: The univariate multivariable logistic regression models for suboptimal adherence were unadjusted models

Multivariable models:

^a^ The multivariate logistic regression model for suboptimal adherence included CTQ total scores, CD-RISC scores, age, sex, marital status, education level, HAMD-17 scores, GAD-7 scores, suicidal ideation, suicidal attempt, insomnia, SSI pain-related items, respectively

^b^ The multivariate logistic regression model for suboptimal adherence included scores of five CTQ dimensions, CD-RISC scores, age, sex, marital status, education level, HAMD-17 scores, GAD-7 scores, suicidal ideation, suicidal attempt, insomnia, SSI pain-related items, respectively

### Associations between childhood trauma and medication adherence by resilience

As shown in Table 3, after adjusting for selected covariates (including age, sex, marital status, education level, HAMD-17 scores, GAD-7 scores, suicidal ideation, suicidal attempt, insomnia, SSI pain-related items), the final multivariable logistic regression analyses demonstrated that the interaction items (CTQ total scores/CTQ of physical neglect scores and CD-RISC scores) with medication adherence were statistically significant (all *P* for interaction < 0.05) (Table 3).

**Table 3:** Association of interaction items with medication adherence among MDD (*N* = 282)

Interaction Item^a^	Suboptimal adherence	
	*AOR* (95%*CI*)	*P* Value
CD-RISC scores_*_		
CTQ total scores	0.97 (0.94–0.99)	**0.039**
CTQ scores of sexual abuse	1.00 (0.99–1.02)	0.597
CTQ scores of physical neglect	0.99 (0.98–1.00)	**0.034**

Abbreviations: *AOR* Adjusted odds ratio, *CI* Confidence interval, *CD-RISC* Connor-davidson resilience scale, *CTQ* Childhood trauma questionnaire-28 item short form

^a^For every 1-score increase in the interaction items between total CTQ scores/CTQ scores of sexual abuse/CTQ scores of physical neglect and CD-RISC scores; the interaction items as well as age, sex, marital status, education level, HAMD-17 scores, GAD-7 scores, suicidal ideation, suicidal attempt, insomnia, and SSI pain-related items were simultaneously entered into the multivariable logistic regression models

According to the percentile of CD-RISC scores, the participants were divided into three groups: low-resilience group (*n* = 60), defined as having CD-RISC score ≤ lower quartile (*Q*_L_); moderate-resilience group (*n* = 143), defined as having CD-RISC score QL and less than upper quartile (*Q*_U_); high-resilience group (*n* = 79), defined as having CD-RISC score ≥ *Q*_U_ [24]. The distribution of medication adherence in the low, medium and high resilience group are presented eFigure 1 in the supplement. After adjusting for selected covariates (including age, sex, marital status, education level, HAMD-17 scores, GAD-7 scores, suicidal ideation, suicidal attempt, insomnia, SSI pain-related items), stratified analyses found that CTQ total scores (*AOR* = 1.01, 95%*CI* = 1.00–1.11, *P* < 0.05) and CTQ scores of physical neglect (*AOR* = 1.05, 95%*CI* = 1.00–1.15, *P* < 0.05) were significantly associated with medication adherence in low-resilience group. The associations of CTQ total scores (*AOR* = 1.06, 95%*CI* = 1.01–1.11, *P* < 0.05) and CTQ scores of physical neglect (*AOR* = 1.05, 95%*CI* = 1.00–1.15, *P* < 0.05) with medication adherence were also found in moderate-resilience group. However, the foregoing associations were not statistically significant among patients with high-resilience. In summary, childhood trauma and physical neglect were associated with an increased risk of suboptimal adherence among patients with low-resilience and moderate-resilience. The foregoing results are summarized in Table 4.

**Table 4:** Association of childhood trauma and medication adherence by Resilience among MDD (*N* = 282)

Variables	Low resilience (*N* = 60)		Moderate resilience (*N* = 143)		High resilience (*N* = 79)	
	*AOR* (95%*CI*)	*P* Value	*AOR* (95%*CI*)	*P* Value	*AOR* (95%*CI*)	*P* Value
CTQ total scores^a^	1.01(1.00–1.11)	**0.049**	1.06 (1.01–1.11)	**0.045**	1.01 (0.99–1.06)	0.978
CTQ scores of physical neglect^a^	1.05(1.00–1.15)	**0.042**	1.05 (1.00–1.15)	**0.042**	1.01 (0.83–1.24)	0.920

Abbreviations: *AOR* Adjusted odds ratio, *CI* Confidence interval, *CTQ* Childhood trauma questionnaire-28 item short form

^a^For every 1-score increase in total CTQ scores, CTQ scores of physical neglect; the multivariate logistic regression models for medication adherence were adjusted for age, sex, marital status, education level, HAMD-17 scores, GAD-7 scores, suicidal ideation, suicidal attempt, insomnia, SSI pain-related items, respectively

## Discussion

Early detection of suboptimal medication adherence and timely intervention among patients with MDD is a priority for individuals receiving pharmacotherapy [52]. To our knowledge, this is the first study to test the prospective association between childhood trauma, resilience, and medication adherence among persons living with MDD.

The study herein used the MARS as the adherence measure, focusing on medication behavior, beliefs, and negative side effects of taking medication. A high percentage of the sample (83%) reported having suboptimal medication adherence. This is not surprising as poor adherence to medication in persons with MDD has been widely reported in the literature [3–5]. Our results indicate higher rates of non-adherence in patients with MDD than rates reported by other studies. The causes of the high prevalence rates may be related to the different measurement methods of suboptimal adherence, and it has been stated that self-report questionnaires tend to overestimate adherence behavior [53], which suggest that the rate of suboptimal adherence found in this study could be even higher. For the reason of high suboptimal adherence, it can be explained by the sample were enrolled at a place specialized in mood disorders, which is probably a population particularly nonadherent to treatment and severely ill, as illustrated for the observation that the severe depression symptomatology, anxious symptomatology, and high proportion of insomnia, and somatic pain [54, 55].

Our study identifies sociodemographic, clinical and psychosocial factors associated with medication adherence through preliminary analysis. We observed that education level, depression severity, anxiety severity, suicidal ideation, suicidal attempt, insomnia symptoms, painful somatic symptoms, childhood trauma (including physical abuse, sexual abuse, and physical neglect), and resilience were associated with medication adherence. The foregoing results were consistent with other previous findings among patients under psychotropics [3, 56]. It can be hypothesized that a vicious circle in which more vulnerable patients (e.g., individuals who are severely depressed, anxious, and having more suicidal thoughts, insomnia symptoms, childhood trauma, painful somatic symptoms, and reduced resilience) are less adherent to medication, which could worsen the clinical picture maintaining, in turn, low adherence [25]. Therefore, early identification of suboptimal adherence should consider behavioral factors susceptible to influence medication adherence when providing MDD pharmacotherapy.

To date, relatively few studies have examined the association between childhood trauma and suboptimal medication adherence with controlling for the effects of relevant factors among persons with MDD [3, 41, 57]. After adjusting for age, sex, marital status, education level, HAMD-17 scores, GAD-7 scores, suicidal ideation, suicidal attempt, insomnia, SSI pain-related items, our study suggests that childhood trauma, especially sexual abuse, as well as physical neglect, are closely associated with medication adherence. In contrast, another study failed to control for confounders argued that emotional abuse was the strongest predictor of suboptimal adherence among patients with MDD [41]. The foregoing differences may be related to the cultural background and traditional education methods of China and western countries, this merit more further research attention.

Childhood trauma may predict medication adherence through neurobiological and social pathway. Compared with individuals who have not experienced childhood trauma, those with a history of childhood trauma are at greater risk of enduring cognitive and biological vulnerabilities associated with heightened stress sensitivity, which might predispose them to suboptimal adherence [17]. In addition, individuals with childhood trauma tend to have a poor social support network and trouble building trust with others [58], therefore, social support network may be a critical factor improving medication adherence. This is particularly significant as having a strong support network, the family’s reaction to the disease, and their ability to support and help the patient to pursue the long course of treatment is a critical factor affecting medication adherence [59].

In addition, our study suggested that resilience may play a moderating role in the association of childhood trauma and physical neglect with medication adherence. Further stratification analyses according to the resilience group indicated that both childhood trauma and physical neglect were associated with an increased risk of suboptimal adherence among patients with MDD with low-resilience or moderate-resilience. However, the associations between childhood trauma/physical neglect and medication adherence were not found among patients with MDD with high-resilience. People with greater resilience are more likely to be good at recovering from difficulties and stressful situations [60, 61]. Resilience buffer against stressful events such as childhood trauma, and weaken the association between childhood trauma and medication adherence [62]. Therefore, the adverse effects of childhood trauma on medication adherence may not be further boosted under the condition of higher resilience. Of course, because of the imbalance of medication adherence in high-resilience group (7.6% VS 92.4%), it may lead to the underpower of the study, and the foregoing associations were not found, this possibility calls for further large sample examinations.

The present results have significant implications for future practice in preventing and managing MDD. Clinicians may consider adding childhood trauma and resilience assessment in their inquiry about during MDD diagnosis, which may provide significant prognostic information to their pharmacotherapy. In addition, even though resilience is generally considered to have trait-like characteristics, resilience capacity can be altered [28]. Enhancing resilience is a method that focuses on enhancing strategies to cope with adherence and diminishing the likelihood of non-adherence [63]. There are studies suggesting that some psychotherapeutic interventions, such as CBT, can effectively enhance resilience [64–66]. Psychiatrists may consider paying attention and giving targeted assistance to improve resilience when providing pharmacotherapy to patients with MDD, especially for individuals with a history of childhood trauma.

Furthermore, childhood is thought to be a sensitive developmental period for the maturation of emotion regulation [67], interventions aimed at reducing childhood trauma could help prevent the large health and economic burden linked to poor depression course [17], and parents should recognize the adverse effects of childhood trauma on their children and give them more physical or psychological care [68].

Major strengths of this study are the prospective design, the use of objective measures widely used in MDD patients (M.I.N.I., HAMD-17, MARS, CTQ-SF, CD-RISC, etc.), and controlling for the effects of factors associated with medication adherence or childhood trauma during statistical analysis, which has better quality of evidence. However, there is limitation to this study. Although most data were measured by self-report, which may lead to biased reporting of childhood trauma experience, resilience, and the adherence behavior can be overestimated, self-reports remain a common and accepted method. In this regard, further studies should combine self-reports with more objective indirect measurements of adherence, such as pill counts and prescription refills [69, 70].

## Conclusion

In conclusion, our results indicate that childhood trauma was significant risk factor of suboptimal adherence among individuals with MDD, and resilience moderated the foregoing association. Based on the findings of our study, the early detection and timely intervention of suboptimal medication adherence by obtaining a history of childhood trauma were considered, particularly for those with lower resilience. In clinical settings, psychiatrists and other healthcare professionals may consider improving medication adherence by methods of enhancing resilience, especially in individuals with a history of childhood trauma.

## Supplementary Information

**Additional file 1:** **eTable 1.** Baseline sample characteristics between eligible and ineligible participants. **eTable** **2.** Frequencies of responses on the MARS among the suboptimal adherence group (*n*=234). **eFigure 1.** The distribution of suboptimal adherence in the low, medium and high resilience group.

## Data Availability

The datasets used and/or analyzed during the current study available from the corresponding author on reasonable request.

## Abbreviations

MDD: Major depressive disorder
M.I.N.I: Mini-international neuropsychiatric interview
CTQ-SF: Childhood trauma questionnaire-28 item short form
CD-RISC: Connor-davidson resilience scale
SCID-I: Structured clinical interviews for DSM-IV
PAR: Population attributable risk
DCC: Depression cohort in China
HAMD-17: The 17-item Hamilton rating scale for depression
MARS: Medication adherence rating scale
DAI: Drug attitude inventory
MAQ: Medication adherence questionnaire
HSS: Household socioeconomic status
GAD-7: Generalized anxiety disorder scale-7
ISI: Insomnia severity index
SSI: Somatic symptoms inventory
SD: Standard deviation
IQR: Interquartile range
AOR: Adjusted odds ratio
CI: Confidence interval
