# Supplementary material for: Association between childhood trauma and medication adherence among patients with major depressive disorder: the moderating role of resilience
Source: BMC Psychiatry. 2022 Oct 14;22:644. doi: 10.1186/s12888-022-04297-0 (PMC9563806; doi:10.1186/s12888-022-04297-0)
Supplement: Supplementary file 1 — Additional file 1: eTable 1. Baseline sample characteristics between eligible and ineligible participants. eTable 2. Frequencies of responses on the MARS among the suboptimal adherence group (n=234). eFigure 1. The distribution of suboptimal adherence in the low, medium and high resilience group. [file 12888_2022_4297_MOESM1_ESM.docx]

eTable1. Baseline sample characteristics between eligible and ineligible participants.

| Variables | Total, *n* (%) | Eligible | Ineligible | *P* Value^#^ |
| --- | --- | --- | --- | --- |
| Total | 344 (100) | 282 (82.0) | 62 (18.0) |  |
| Age (years), median (*IQR*) | 25.0 (9.0) | 25.0 (9.0) | 25.5 (8.0) | 0.968 |
| Sex |  |  |  |  |
| Male | 109 (31.7) | 95 (33.7) | 14 (22.6) | 0.089 |
| Female | 235 (68.3) | 187 (66.3) | 48 (77.4) |  |
| Marital status |  |  |  |  |
| Unmarried | 243 (73.2) | 199 (73.2) | 44 (73.3) | 0.975 |
| Married | 74 (22.3) | 61 (22.4) | 13 (21.7) |  |
| Divorced/Widowed | 15 (4.5) | 12 (4.4) | 3 (5.0) |  |
| Missing data |  |  |  |  |
| Education level |  |  |  |  |
| Junior high school or below | 60 (17.8) | 50 (18.1) | 10 (16.1) | 0.903 |
| Senior high school | 92 (27.2) | 74 (26.8) | 18 (29.0) |  |
| College or above | 186 (55.0) | 152 (55.1) | 34 (54.8) |  |
| Missing data |  |  |  |  |
| Living arrangement |  |  |  |  |
| Living alone | 78 (23.4) | 61 (22.3) | 17 (28.3) | 0.192 |
| Living with families | 193 (57.8) | 167 (60.9) | 26 (43.3) |  |
| Living with others | 63 (18.9) | 46 (16.8) | 17 (28.3) |  |
| Missing data |  |  |  |  |
| HSS |  |  |  |  |
| Excellent or very good | 130 (42.2) | 106 (41.6) | 24 (45.3) | 0.248 |
| Good | 92 (29.9) | 73 (28.6) | 19 (35.8) |  |
| Fair | 86 (27.9) | 76 (29.8) | 10 (18.9) |  |
| Missing data |  |  |  |  |
| Current cigarette smoking |  |  |  |  |
| No | 158 (47.0) | 130 (47.3) | 28 (45.9) | 0.846 |
| Yes | 178 (53.0) | 145 (52.7) | 33 (54.1) |  |
| Missing data |  |  |  |  |
| Current alcohol drinking |  |  |  |  |
| No | 292 (85.6) | 237 (84.9) | 55 (88.7) | 0.445 |
| Yes | 49 (14.4) | 42 (15.1) | 7 (11.3) |  |
| Missing data |  |  |  |  |
| Exercise habit per week |  |  |  |  |
| No | 211 (53.4) | 171 (63.1) | 40 (64.5) | 0.901 |
| Yes | 122 (36.6) | 100 (36.9) | 22 (35.5) |  |
| Missing data |  |  |  |  |
| HAMD-17 scores, mean (*SD*) | 17.86 (5.19) | 17.85 (5.09) | 17.93 (5.64) | 0.901 |
| GAD-7 scores, median (*IQR*) | 14.0 (8.0) | 14.0 (8.0) | 14.0 (8.0) | 0.792 |
|  |  |  |  |  |
|  |  |  |  |  |
| eTable 1. Baseline sample characteristics between eligible and ineligible participants. (continued). | | | | |
| Suicidal ideation |  |  |  |  |
| No | 234 (68.6) | 192 (68.8) | 42 (67.7) | 0.869 |
| Yes | 107 (31.4) | 87 (31.2) | 20 (32.2) |  |
| Missing data |  |  |  |  |
| Suicidal attempt |  |  |  |  |
| No | 299 (87.4) | 249 (88.9) | 50 (80.6) | 0.075 |
| Yes | 43 (12.6) | 31 (11.1) | 12 (19.4) |  |
| Missing data |  |  |  |  |
| Insomnia |  |  |  |  |
| None | 52 (15.2) | 40 (14.3) | 12 (19.4) | 0.549 |
| Subthreshold | 104 (30.5) | 85 (30.5) | 19 (30.6) |  |
| Moderate | 128 (37.5) | 109 (39.1) | 19 (30.6) |  |
| Severe | 57 (16.7) | 45 (16.1) | 12 (19.4) |  |
| Missing data |  |  |  |  |
| SSI pain-related items |  |  |  |  |
| Without pain | 108 (31.5) | 90 (31.9) | 18 (29.5) | 0.714 |
| With pain | 235 (68.5) | 192 (68.1) | 43 (70.5) |  |
| CD-RISC scores, median (*IQR*) | 37.0 (18.0) | 37.0 (18.0) | 38.0 (19.0) | 0.698 |
| CTQ total scores, median (*IQR*) | 46.0 (16.0) | 46.0 (16.0) | 46.0 (18.0) | 0.697 |
| CTQ scores of physical abuse, median (IQR) | 5.0 (3.0) | 6.0 (3.0) | 5.0 (4.0) | 0.261 |
| CTQ scores of emotional abuse, median (IQR) | 9.0 (5.0) | 9.0 (5.0) | 8.0 (6.0) | 0.388 |
| CTQ scores of sexual abuse, median (IQR) | 5.0 (0.0) | 5.0 (0.0) | 5.0 (1.0) | 0.225 |
| CTQ scores of physical neglect, median (IQR) | 9.0 (5.0) | 8.0 (5.0) | 9.0 (5.0) | 0.648 |
| CTQ scores of emotional neglect, median (IQR) | 16.0 (8.0) | 15.0 (8.0) | 16.0 (9.0) | 0.711 |

Abbreviations: SD, standard; HSS, household socioeconomic status; HAMD-17, 17-item Hamilton Rating Scale for Depression; GAD-7, the Generalized Anxiety Disorder Scale-7; SSI, Somatic Symptoms Inventory; CD-RISC, Connor-Davidson Resilience Scale; CTQ, Childhood Trauma Questionnaire-28 item Short Form.

^#^Chi-squared tests were used for categorical variables, *t* test was used for HAMD-17 scores data, and Wilcoxon’s rank-sum tests were used for other continuous data.

eTable 2. Frequencies of responses on the MARS among the suboptimal adherence group (n=234).

| Items | | Yes | No |
| --- | --- | --- | --- |
|  |  | n (%) | n (%) |
| 1 | I sometimes forget to take my medication | 117 (50.0) | 117 (50.0) |
| 2 | I'm careless at times about taking my medication | 117 (50.0) | 117 (50.0) |
| 3 | If I feel better, sometimes I stop the treatment | 170 (72.6) | 64 (27.4) |
| 4 | Sometimes if I feel worse when I take my medication, I stop taking it | 207 (88.5) | 27 (11.5) |
| 5 | I take my medication only when I am sick | 180 (76.9) | 54 (23.1) |
| 6 | It's not natural for my body and mind to be balanced by drugs | 180 (76.9) | 54 (23.1) |
| 7 | My ideas are clearer with medication | 129 (55.1) | 105 (44.9) |
| 8 | By staying on medication, I can prevent getting sick | 112 (47.9) | 122 (52.1) |
| 9 | With the drugs I feel weird, like a zombie | 192 (82.1) | 42 (17.9) |
| 10 | The drugs make me heavy and tired | 147 (62.8) | 87 (37.2) |


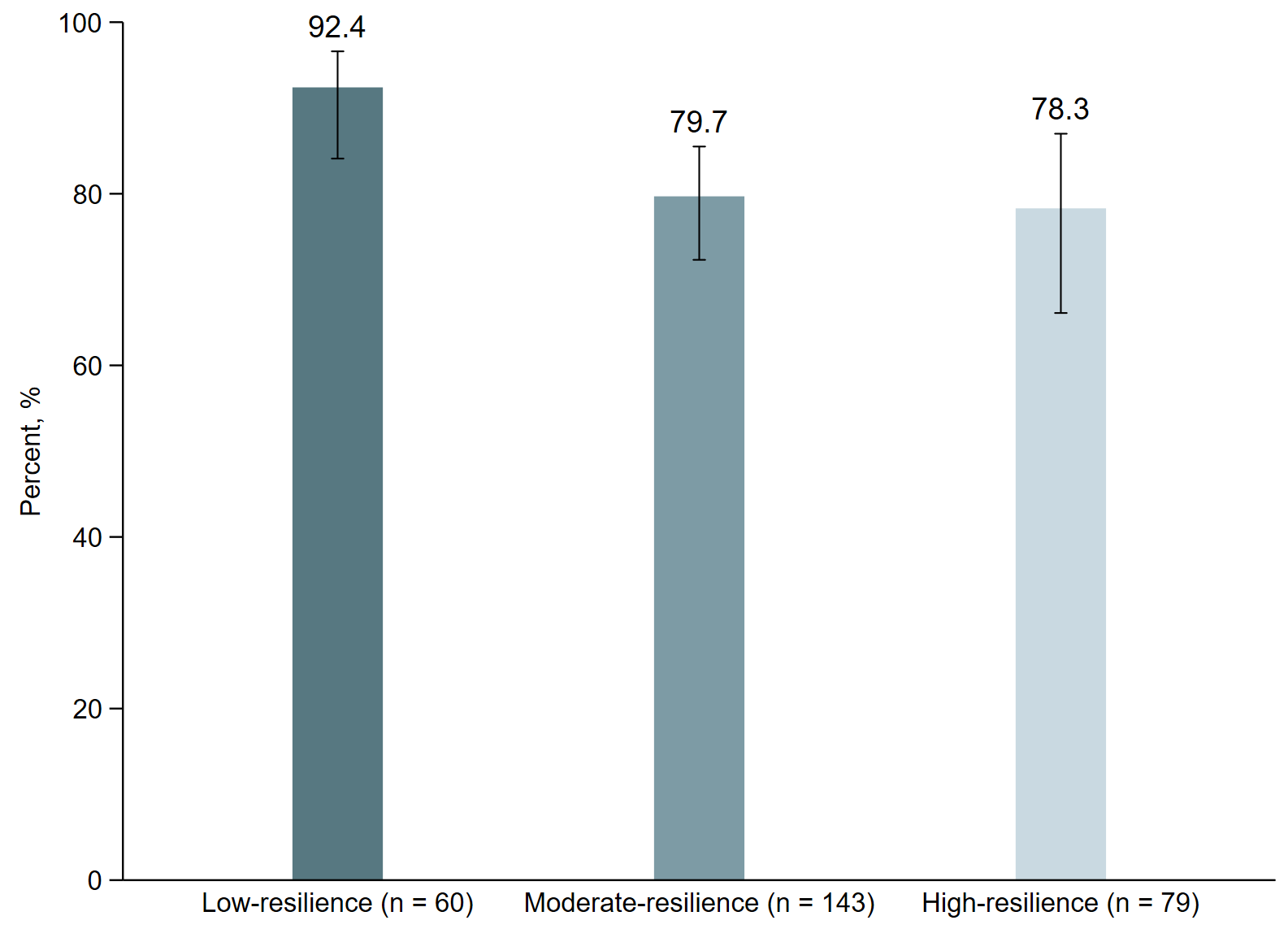


eFigure 1. The distribution of suboptimal adherence in the low, medium and high resilience group.
